# Efficiency and Stability of Transarterial Chemoembolization Combined With or Without Lenvatinib for Unresectable Hepatocellular Carcinoma

**DOI:** 10.5152/tjg.2024.23071

**Published:** 2024-03-01

**Authors:** Zheng Zong, Rongyu Tang, Mingyu Li, Xinmiao Xiong, Daixin Li, Jing Fan, Wei Ye, Chenqi Xue

**Affiliations:** 1 Department of Infectious and Liver Disease, The Second Hospital of Nanjing, Affiliated to Nanjing University of Chinese Medicine, Nanjing, China; 2 Clinical Research Center, The Second Hospital of Nanjing, Affiliated to Nanjing University of Chinese Medicine, Nanjing, China

**Keywords:** Lenvatinib, unresectable hepatocellular carcinoma, transarterial chemoembolization, overall survival, progression-free survival, adverse events

## Abstract

**Background/Aims:**

At present, there are relatively few reports on the treatment consisting of transarterial chemoembolization (TACE) combined with lenvatinib, and there is no unified conclusion on the curative effect. The objective of this research was to assess the efficacy and safety of combining TACE with lenvatinib for the treatment of unresectable hepatocellular carcinoma (uHCC).

**Materials and Methods:**

This study was a retrospective analysis of the patient’s medical records. In this study, 249 patients (uHCC) in our hospital from 2020 to 2021 were divided into 2 groups, including the TACE-alone group (198 patients received TACE alone) and the TACE-LEN group (51 patients were treated with TACE combined with lenvatinib). According to the propensity score matching method, there were TACE-LEN group (51 patients) and TACE-alone group (51 patients). With the help of surgical experts, the overall survival (OS), progression-free survival (PFS), and tumor response (according to mRECIST) of the 2 groups were sorted and recorded, and then analyzed. Survival curves were established, the prognostic factors of OS and PFS were analyzed by univariate and multivariate analyses, and the independent prognostic factors were recorded. The adverse reactions of patients after treatment were recorded.

**Results:**

The 1-year and 2-year OS rates were 50.98% and 19.48% for the TACE-LEN group, 27.45% and 8.55% for the TACE-alone group (*P* = .042), respectively. The PFS of patients in the TACE-LEN group was also longer (1-year PFS rate: 25.49% vs. 11.76%, 2-year PFS rate: 19.17% vs. 5.88%; *P* = .0069). The disease control rate (68.63% vs. 49.10%, *P* = .044) of the TACE-LEN group was significantly higher. In the subgroup analysis, the OS of the TACE-LEN group was better than TACE-alone group in patients with Barcelona Clinic Liver Cancer stage C (1-year OS rate: 44.44% vs. 17.14%, 2-year OS rate: 8.67% vs. 0%; *P* = .009). Factor analysis concluded that serum alkaline phosphatase and treatment protocol (TACE-LEN vs. TACE) were independent influencing factors of OS. The most common treatment-related AEs included decreased albumin (n = 28, 54.9%), hypertension (n = 23, 45.1%), elevated aspartate transaminase (n = 21, 41.2%) and elevated total bilirubin (n = 18, 35.2%) in TACE-LEN group.

**Conclusion:**

Compared with TACE monotherapy, TACE combined with lenvatinib effectively prolonged the OS time with a controllable safety profile for patients with uHCC.

**Main Points**

- According to the propensity score matching method, there were transarterial chemoembolization (TACE) combined with lenvatinib (TACE-LEN) group (51 patients) and TACE-alone group (51 patients). Patients in the 2 groups were followed up.
- The overall survival (OS), progression-free survival, and disease control rate was higher among patients of the TACE-LEN than the TACE alone.
- In the subgroup analysis, the OS of the TACE-LEN group was better than that of the TACE-alone group in patients with Barcelona Clinic Liver Cancer stage C (1-year OS rate: 44.44% vs. 17.14%, 2-year OS rate: 8.67% vs. 0%; *P* = .009). Serum alkaline phosphatase levels and the choice of treatment (TACE-LEN vs. TACE) were found to be independent predictors of OS.
- The most frequently reported treatment-related AEs included decreased albumin (n = 28, 54.9%), hypertension (n = 23, 45.1%), elevated aspartate transaminase (n = 21, 41.2%), and elevated total bilirubin (n = 18, 35.2%) in the TACE-LEN group.

## Introduction

At present, hepatocellular carcinoma (HCC) accounts for more than 85% of the pathological classifications of primary carcinoma of the liver. This is an important cause of cancer-related deaths in humans, and it also represents the third highest cancer death rate in China.^1^ At present, good results have been achieved in medical, local, and surgical treatment, and the prognosis of patients with early HCC has improved. However, most patients have advanced HCC when they are diagnosed, and their treatment and prognosis are still not significantly improved.^2,3^

Transarterial chemoembolization (TACE) is a recognized clinical method for the treatment of advanced HCC.^4^ Several studies have displayed the advantages of TACE being less invasive, having a wide application range, and light adverse reactions, and prolonging the OS of Liver cancer patients, even for Barcelona Clinic Liver Cancer (BCLC) C patients.^5-8^ After TACE alone, although most of the blood supply of the hepatic artery can be blocked, the portal vein will continue to supply blood to the tumor, and the establishment of arteriovenous collateral branches can make the tumor continue to grow.^9^ Transarterial chemoembolization alone also stimulates the expression of vascular endothelial growth factor (VEGF), which can stimulate tumor neovascularization and revascularization, ultimately leading to tumor recurrence.^10^ Therefore, how to develop new therapeutic strategies for unresectable HCC (uHCC) patients, especially those with poor response to TACE, still needs to be explored.

Adding antiangiogenic agents to transcatheter arterial embolization (TAE) could inhibit the angiogenesis stimulated by TAE, synergize with TAE in suppressing the growth of tumors and prolong survival in rat experiments.^11^ Some studies also provided better experimental evidence that TACE+sorafenib can improve the OS of patients with uHCC and is well tolerated compared with TACE alone.^12,13^ In a multicenter prospective trial, the mPFS of patients receiving TACE + sorafenib was 25.6 months, which was significantly longer than the TACE-alone group (*P* = .006).^14^

Lenvatinib, as a new first-line oral multikinase inhibitor, has been widely used in the clinical treatment of liver cancer. Lenvatinib has antiangiogenic and direct antitumor effects by targeting multiple kinase receptors, including VEGF, fibroblast growth factor (FGF), and platelet-derived growth factor receptors.^15,16^ Compared with sorafenib, lenvatinib treatment significantly improved the PFS and disease control rate of patients. In addition, lenvatinib has a relatively high objective remission rate (ORR) (33.3% vs. 6.5%) and disease control rate (DCR) (76.9% vs. 52.7%).^17,18^

At present, there are relatively few reports about the treatment of lenvatinib combined with TACE, and there is no consistent conclusion about the efficacy. Therefore, the efficacy and adverse reactions of TACE plus lenvatinib compared with TACE monotherapy in the treatment of uHCC were retrospectively analyzed in our study.

## Materials and Methods

### Patients

We collected and screened patients who were diagnosed with uHCC at the Second Hospital of Nanjing from June 2020 to June 2021 and then performed a retrospective analysis. According to the Guidelines of the American Association for the Study of Liver Diseases,^19^ hepatocellular carcinoma was diagnosed by biopsy, cytology, dynamic computed tomography (CT), or magnetic resonance imaging. The tumor staging of the patient was performed by the surgeon according to the Barcelona Liver Cancer staging system (BCLC) and the China liver cancer staging system (CNLC).

Requirements for patients to be grouped: HCC was diagnosed by histopathological biopsy or according to clinical features; uHCC was defined as those with assessment of BCLC B or BCLC C stages due to insufficient liver reserve function, multifocal tumors, extrahepatic metastases, and large vessel invasion; Eastern Tumor Cooperative group presentation status 0 or 1; Child‒Pugh class A or B; according to mRECIST, there was at least one measurable targeted lesion; no systematic treatment for HCC before. Conditions for patients to be deleted were as follows: live cancer caused by tumor metastasis from other organs; patients who also received other forms of treatment, such as immune checkpoint inhibitors, radiation, and ablation; there were also other primary malignancies; Child‒Pugh class C. The research plan was approved by the Ethics Committee of the Second Hospital of Nanjing (Approval No: 2020-LY-k1081, Date: 01.05.2020). No informed consent was needed because of the retrospective non-interventional study design.

### TACE Therapy

The Seldinger technique was used to insert a catheter through the femoral artery into the hepatic artery, followed by hepatic angiography. The interventional physician selectively inserted the catheter tip into the tumor-feeding artery under CT guidance. Then, pirarubicin emulsified with iodide was injected into the blood vessel. Finally, granular embolic materials were selectively added to ensure satisfactory embolization of the feeding artery. Post-TACE Assessment was conducted every 6 to 8 weeks, and TACE was repeated if necessary.

### Lenvatinib Therapy

Lenvatinib therapy should be conducted as follows: patients in the TACE-LEN were already taking lenvatinib after enrollment. Before recommending TACE plus lenvatinib, patients should fully understand the efficacy, adverse reactions that may occur during treatment, and costs of the drug. Lenvatinib was given within 3 days of the first TACE treatment if the patient agreed with the doctor‘s advice on medication. Lenvatinib was given once a day, and the specific dosage depended on the body weight. The dosages of lenvatinib were 12 mg (weight ≥60 kg) and 8 mg (weight <60 kg). The patient should stop taking lenvatinib for 3 days before each TACE treatment, and clinical observation is required after TACE. If adverse event (AE) induction (such as fever, nausea, and vomiting) did not occur, the patient could resume taking lenvatinib after TACE treatment. If the adverse reaction caused by TACE was serious, patients should stop taking lenvatinib until their adverse reaction was relieved. According to the guidelines, it was permissible to interrupt the dose of lenvatinib to reduce the toxicity associated with lenvatinib (to 8 mg and 4 mg daily or 4 mg every other day). Patients taking lenvatinib did not take other antitumor drugs.

### Efficacy Assessment

The primary endpoint for therapeutic efficacy evaluation was OS. The secondary endpoints of other therapeutic efficacy evaluations were PFS, ORR, and safety. Compared with the traditional RECIST evaluation criteria, mRECIST evaluation criteria could more accurately and comprehensively evaluate the efficacy of interventional therapy and molecular targeted ttherapy for HCC patients. According to mRECIST, OS was the time from the start of treatment to their death. PFS was the time from the start of treatment until tumor progression or their death from any cause occurs.

### Safety Assessment

The safety of treatment-emergent AEs was evaluated primarily in accordance with the Standard for Common Terminology for Adverse Events (CTCAE, Version 5.0). We recorded all AEs, and the follow-up interval was 6-8 weeks. Post-TACE-related AEs, such as abdominal pain and fever, were not recorded.

### Antiviral Therapy

All hepatitis B patients (HBV) should be treated with antiviral drugs under the clinician‘s guidance prior to treatment and as long-term treatment. Changes in viral load levels were also monitored during patient follow-up. Under the guidance of clinicians, hepatitis C patients (HCV) should also r should be treated with antiviral drugs from the beginning of treatment.

### Propensity Score Matching Analysis

We used a logistic regression model to fit the 3 relevant variables Barcelona Clinical Liver Cancer (BCLC), Child‒Pugh classification, and China Liver Cancer Staging (CNLC) for propensity score analysis as recommended by the clinicians. We used nearest neighbor matching (1 : 1 propensity matching) to establish a propensity-matched cohort for the TACE-LEN and the TACE-alone.

### Statistical Analysis

General characteristics, tumor response, and AEs were compared using a *t* test, Fisher’s exact test, or the chi-square test. According to the follow-up data of patients, the Kaplan‒Meier method was used to draw the survival curve, and the log-rank test was used for significance analysis. Univariate and multivariate analyses were conducted based on the Cox regression model. All statistical analysis used Statistical Package for the Social Sciences Statistics (version 22.0, IBM Corp.; Armonk, NY, USA) and GraphPad Prism (version 8.0.2), and *P* < .05 was significant.

## Results

### General Characteristics of the Patients

**Figure 1.**
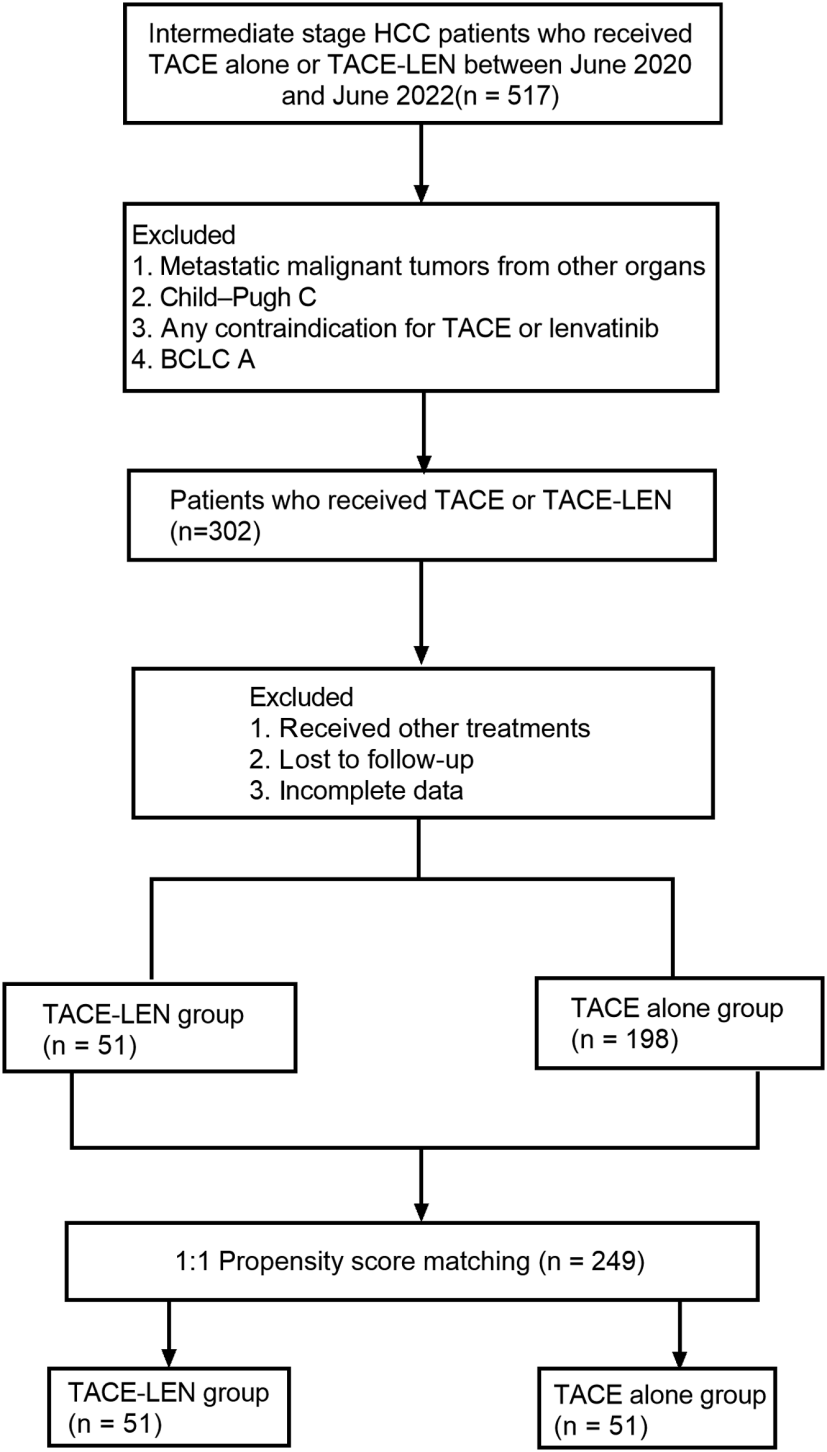
Flow diagram of patient enrollment.

The patient registration flowchart was shown in Figure 1. From June 2020 to June 2021, 517 patients with unresectable liver cancer were analyzed retrospectively. Of these, 215 patients were deleted according to the admission criteria. The combination of other treatments, no follow-up, and loss of information were excluded. The remaining 302 patients were included, with 51 patients receiving TACE-LEN and 198 patients receiving TACE alone. The 302 patients were matched with propensity score to obtain 2 groups, 51 patients receiving TACE-LEN and 51 patients receiving TACE alone. The data of patients at baseline were listed in Table 1. Both groups achieved a good balance in baseline characteristics, including sex, age, Child‒Pugh grade, etiology of HCC, BCLC stage, AFP level, prothrombin time (PT), ALBI, albumin (ALB), total bilirubin (TB), Model for End-stage Liver Disease (MELD) score, platelet count (PLT), extrahepatic metastasis, and vascular invasion. The average number of TACE treatments for patients in the TACE-LEN group was 2.92, while the TACE-alone group was 3.75. The number of treatments in the TACE alone group was slightly higher, but there was no statistical significance.

### Tumor Responses

Based on the mRECIST guidelines, the proportions of patients with CR, PR, SD, and PD in the TACE-LEN were 3.9%, 29.4%, 35.3%, and 31.4%. The ORR (CR+PR) was 33.33%. The DCR (CR + PR + SD) was 68.63%. In contrast, in the TACE-alone group, CR was 5.9%, PR was 19.6%, SD was 23.5%, and PD was 51%. ORR was 25.5%. The DCR was 49.1%. The DCR of the TACE-LEN was higher compared with that of the TACE alone (68.63% vs. 49.10%, *P* = .044). However, the ORR (*P* = .128) was not significantly different, as shown in Table 2.

### Survival Assessment

**Figure 2.**
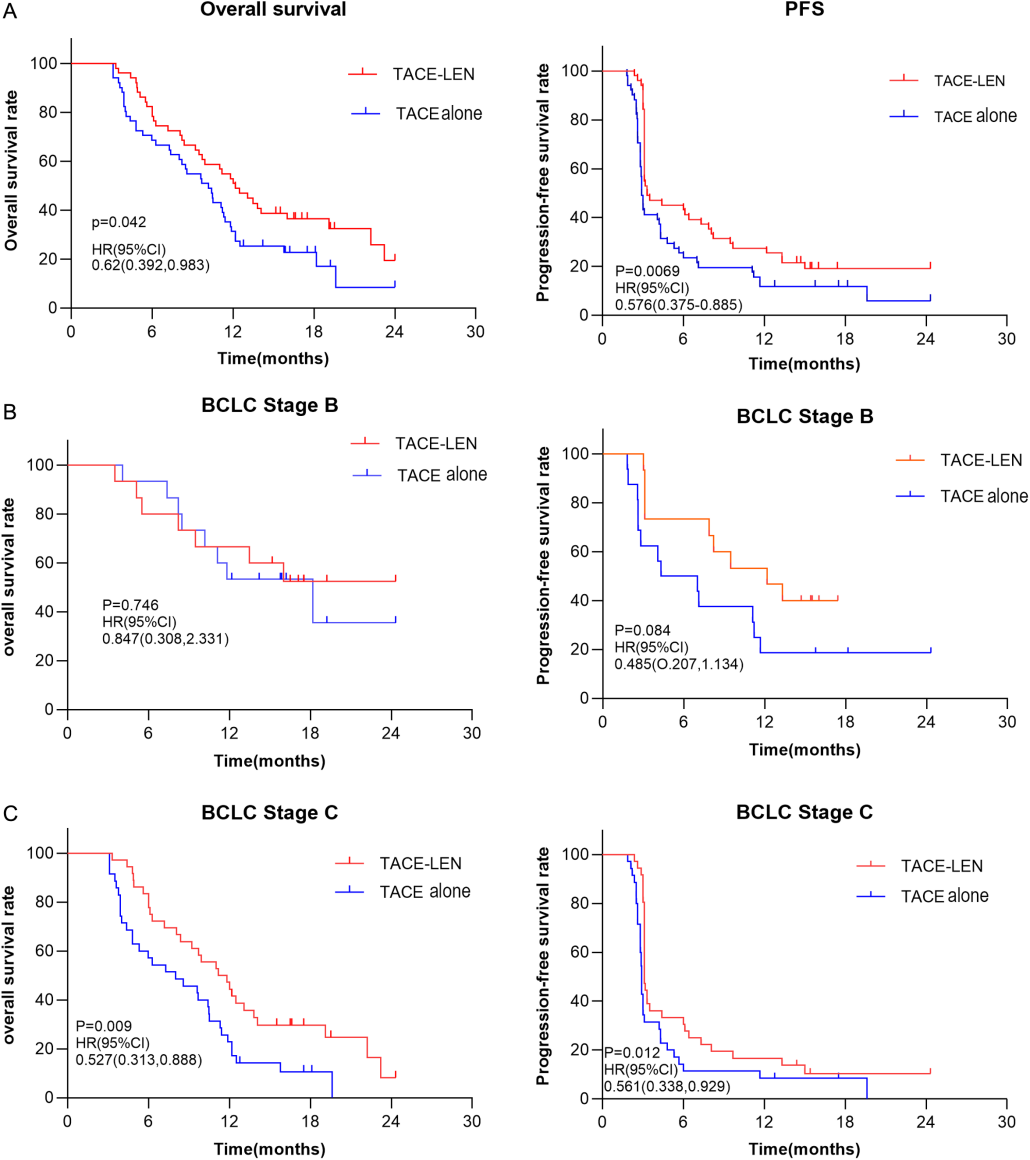
Overall survival and progression-free survival rate with different therapies. (A) OS and PFS in the total population. (B) OS and PFS in BCLC stage B patients. (C) OS and PFS in BCLC stage C patients. BCLC, Barcelona Clinic Liver Cancer; OS, overall survival; PFS, progression-free survival.

As of our last follow-up date (June 10, 2022), there were 35 deaths (TACE-LEN) and 41 deaths (TACE alone). As shown in Figure 2, in this study, patients in the TACE-LEN had better PFS and OS. The 1-year and 2-year OS rates were 50.98% and 19.48% for the TACE-LEN and 27.45% and 8.55% for the TACE alone (*P* = .042; Figure 2A). The mOS was significantly higher in the TACE-LEN (12.17 months vs. 10.17 months; *P* = .042; Table 3). PFS was also longer in the TACE-LEN (1-year PFS rate: 25.49% vs. 11.76%, 2-year PFS rate 19.17 vs. 5.88%; *P* = .0069; Figure 2A). The mPFS of the TACE-LEN group was 3.33 months, and that of the TACE alone was 2.9 months (*P* = .007), as shown in Table 3.

In addition, we analyzed the effects of treatment schemes for patients with BCLC stages B and C. As shown in Figure 2B, OS and PFS were not statistically significant between the 2 groups in BCLC stage B. However, the OS and PFS in the TACE-LEN were longer in BCLC stage C, as shown in Figure 2C and Table 3.

### Analysis of Prognostic Factors Affecting PFS and OS

Univariate analysis showed a significant association of OS with ALP, AFP, hepatic venous invasion, and treatment option (TACE-LEN vs. TACE), as shown in Table 4. Multi-factor analysis shows that the independent prognostic factors of OS were ALP (HR = 1.002, *P* = .039) and treatment plan (TACE-LEN vs. TACE alone) (HR = 2.203, *P* = .002).

Univariate analysis showed that aspartate transaminase (AST), hepatic vein invasion, BCLC stage, and treatment option (TACE-LEN and TACE alone) were significantly correlated with PFS. Multifactor analysis shows that the independent prognostic factors of PFS was treatment option (TACE-LEN vs. TACE alone) (HR = 1.694, *P* = .017), as shown in Table 5.

### Safety Evaluation

A review of all recorded AEs revealed that these AEs were effectively controlled, and no treatment-related deaths were found. As listed in Table 6, the most common AEs of lenvatinib were decreased albumin (n = 28, 54.9%), hypertension (n = 23, 45.1%), increased AST (n = 21, 41.2%), and increased TB (n = 18, 35.2%). The frequently reported grade 3/4 AEs included hypertension (n = 13, 25.5%) and proteinuria (n = 11, 21.6%).

Adverse events recorded in the TACE group were elevated AST (n = 20, 39.2%) and elevated ALT (n = 18, 35.3%).

## Discussion

In this study, the efficacy of combination therapy (TACE-LEN) and TACE alone in the treatment of uHCC was retrospectively analyzed. Our results showed that combination therapy (TACE-LEN) could effectively prolong the OS of patients. The treatment option could be an independent predictor of improvement in our patient‘s prognosis. Taken together, these results suggested that TACE plus lenvatinib could offer greater benefits than TACE alone in patients with uHCC.

Due to the complexity and particularity of uHCC, no optimal therapeutic strategy has been found in clinical practice. At present, regarding the treatment of uHCC, the related treatments are mainly adopted to reduce the staging of liver cancer and slow the progression to the advanced stage of liver cancer to prolong the survival time.^4^ Many clinical practice guidelines recommend TACE as the standard of care for patients with intermediate HCC.^6,7,20^ However, studies show that the efficacy of TACE alone in the treatment of unresectable liver cancer is still limited, and the efficacy and disease control duration of clinical patients has not reached a good level. Undeniably, TACE increases the ischemia and hypoxia of tumor cells, which leads to the upregulation of VEGF and FGF expression and ultimately promotes tumor angiogenesis and tumor growth. Some studies have suggested that TACE combined with systemic therapy (such as targeted drugs) may partly solve this problem.^10,11^ Several real-world studies have shown that TACE + sorafenib could improve the ORR and DCR of patients with advanced liver cancer.^21^

According to the Chinese guidelines for the diagnosis and treatment of primary liver cancer (2019 edition), systemic antitumor therapy, TACE treatment and radiotherapy could be adopted for patients with CNLC IIIb, that is, patients with extrahepatic metastasis. Patients who could receive TACE treatment according to the Chinese guidelines included patients with CNLC Ia, Ib, and IIa HCC who had indications for surgical resection or ablation but were unable or unwilling to undergo surgical resection or ablation due to nonsurgical reasons such as old age, insufficient liver function reserve, and high-risk tumor location. TACE can also be performed in some patients with CNLC IIb, IIIa, and IIIb HCC.^6,7^ As a result, TACE therapy is widely used, even in some late-stage HCC cases in China.

After years of clinical research, a variety of targeted drugs have been approved for the systemic treatment of advanced liver cancer, providing more options for the combination of TACE and targeted drugs. According to the REFLECT trial, the efficacy of lenvatinib was superior to that of sorafenib, mainly reflected in the OS and PFS of patients with HCC. This encouraging result provided a major breakthrough in the treatment of HCC. Lenvatinib is a receptor tyrosine kinase inhibitor that inhibits VEGF receptor kinase activity (targeting VEGFR-1, 2, 3) and may inhibit the upregulation of angiogenic factors after TACE. Previously, lenvatinib has been defined as the first-line drug of uHCC in the treatment of liver cancer by guidelines.^15,16^ Therefore, lenvatinib combined with TACE may inhibit tumor growth by inhibiting the activity of tumor angiogenesis-related factors.^17,18^ Our study showed that TACE-LEN treatment significantly prolonged OS and PFS compared with TACE alone, which was consistent with related studies.^22^

There was no statistical significance in ORR between the TACE and combination groups in this study (*P* = .657), and the mPFS in this study (3.33 months) was significantly lower than those in other studies, such as Chen et al,^23^ which showed mPFs of 6.15 months.^22^ This result may be explained by a higher percentage of BCLC C patients in our study, including 36 cases (70.59%) in the TACE-LEN and 35 cases (68.63%) in the TACE alone.

Recently, a multicenter, randomized, controlled phase III trial (LAUNCH) to evaluate the efficacy and safety of lenvatinib combined with TACE versus lenvatinib alone as first-line treatment for advanced HCC attracted widespread attention. Their research showed that lenvatinib plus TACE could effectively promote the clinical outcomes of patients with uHCC with good safety and was expected to become a new first-line treatment option for patients with advanced HCC.^24^ Kudo’s research also agrees that starting to use lenvatinib early in the treatment of liver cancer may help improve the outcome of some TACE treatments. However, there were still differences in the timing of the combination of TACE with systemic drug therapy in reported clinical trials.^25,26^ Therefore, more clinical studies on the treatment of advanced liver cancer are needed in the future to confirm our results and further solve the problem of combined treatment.

In our study, ALP and treatment (TACE-LEN vs. TACE) were independent predictors of OS based on multivariate analysis. The relationship between serum alkaline phosphatase (ALP) levels and prognosis in patients with hepatocellular carcinoma might be explained by multiple mechanisms. First, increased ALP might suggest that the biliary system was invaded or compressed by the enlarged liver tumor, which represented the late stage of the tumor. Second, ALP can promote tumorigenesis by altering cell cycle regulation and cell proliferation.^27,28^ Third, clinical research shows that ALP levels can be increased in some nonmalignant diseases related to inflammation, such as chronic hepatitis, choledocholithiasis, cholangitis, and pancreatitis.^29^ Because inflammation can be used as a clinical sign of cancer, an increase in ALP may lead to the occurrence and development of cancer.^30^ Fourth, ALP is widely regarded as a sign of tumor metastasis, especially bone metastasis. Therefore, the increase in ALP may be the potential process leading to the poor outcome of HCC patients.^31^

No death cases or grade 4 adverse reactions related to treatment methods were observed in our study. The highest incidence of adverse reactions after treatment with lenvatinib was decreased albumin, and the most common adverse reactions after TACE were increased AST and ALT. Although all patients experienced different hepatic abnormalities after TACE, the hepatic abnormalities were clinically observed to be transient and reversible. Lenvatinib-related AEs were hypertension, decreased albumin, fatigue, elevated AST, elevated TB, decreased PLT, decreased WBC, decreased appetite, proteinuria, diarrhea, and edema. These AEs can be reduced or managed with dose adjustment or discontinuation or symptomatic treatment.

In the evaluation of our results, we must also consider some shortcomings and potential shortcomings of our study. First, our study is a retrospective study, which led to a number of factors that could bias survival results. The willingness of patients to choose lenvatinib could have a potential impact on treatment outcomes. Second, there are not enough patient samples included in this study, so our research results need to be further verified in future large-sample clinical studies. Third, the cohort of patients with uHCC is heterogeneous. Subgroup analyses of patients with BCLC stage C revealed trends in their survival benefits that were generally consistent with the overall patient trends in this study. Subgroup analyses for BCLC stage B patients showed no significant differences. This may be due to the small number of cases in this study and the imbalance in clinical features across subgroups. In addition, to ensure more convincing results, a patient treatment group who took lenvatinib alone should be set up as the control group for TACE combined with lenvatinib.

In conclusion, compared with TACE alone, TACE in combination with lenvatinib can effectively prolong the OS of patients with uHCC with a controllable safety profile.

## Figures and Tables

**Table 1.** Baseline Demographic and Clinical Characteristics of the Patients

Characteristic	TACE-LEN Group (n = 51)	TACE Group (n = 51)	*P*
Gender	3	3	.715
Female	3 (5.88%)	5 (9.8%)	3
Male	48 (94.12%)	46 (90.2%)	3
Age (years)	3	3	.898
<65	42 (82.35%)	44 (86.27)	3
≥65	9 (17.65%)	7 (13.73)	3
Child‒Pugh	3	3	.407
A	31 (60.78%)	35 (68.63%)	3
B	20 (39.22%)	16 (31.37%)	3
Etiology	3	3	.4
HBV	45 (88.23%)	47 (92.16%)	3
HCV	2 (3.92%)	0 (0%)	3
Without	4 (7.85%)	4 (7.84%)	3
AFP (ng/mL)	3	3	.887
<400	26 (50.98%)	31 (60.78%)	3
≥400	25 (49.02%)	20 (39.22%)	3
ALBI grade	3	3	.133
1	8 (15.69%)	19 (37.26%)	3
2	36 (70.59%)	29 (56.86%)	3
3	7 (13.72%)	3 (3.88%)	3
BCLC	3	3	.83
B	15 (29.41%)	16 (31.37%)	3
C	36 (70.59%)	35 (68.63%)	3
CNLC	3	3	0.547
IIb	17 (33.33%)	16 (31.37%)	3
IIIa	16 (31.37%)	21 (41.18%)	3
IIIb	18 (35.3%)	14 (27.45%)	3
MELD	5.65 (−0.16 to 15.9)	5.4 (−0.248 to 19.6)	.213
HGB (g/L)	136.02 ± 15.93	133.53 ± 19.85	.165
AST (U/L)	39.5 (7.9-160.8)	28 (10.4-243.5)	.677
ALT (U/L)	45.3 (14-646)	36.9 (15-248)	.186
PLT (× 10^9^/L)	112 (27-487)	112 (31-227)	.51
TB (µmol/L)	3	3	0.455
≤17.1	18 (35.29%)	18 (35.29%)	3
>17.1	33 (64.71%)	33(64.71%)	3
Albumin (g/L)	3	3	.978
<35	13 (25.49%)	12 (23.53%)	3
≥35	38 (74.51)	39 (76.47%)	3
Cre (µmol/L)	67(38-121)	70(40-285)	0.31
CA19-9 (IU/mL)	17.05 (2-1000)	15.35 (2-195)	.45
ALP (U/L)	129 (45-1052)	106 (38-325)	.631
GGT (IU/L)	117 (18-578)	88 (16.3-737)	.576
PT (seconds)	10.9 (9.2-20.1)	12.4 (9.2-22.9)	.775
PTA (%)	81.76 ± 16.68	76.33 ± 17.98	.795
INR	1.09 (0.9-1.62)	1.16 (0.89-2.27)	.213
Vascular invasion	3	3	.427
Yes	25 (49.02%)	29 (56.86%)	3
No	29 (50.98%)	22 (43.14%)	3
Extrahepatic metastasis	3	3	.29
Yes	19 (37.25%)	14 (27.45%)	3
No	32 (62.75%)	37 (72.55%)	3
Number of TACE treatments	2.92 ± 2.03	3.75 ± 1.97	.503

AFP, alpha-fetoprotein; ALB, albumin; ALBI grade, albumin-bilirubin grade; ALP, alkaline phosphatase; AST, aspartate transaminase; BCLC, Barcelona Clinic Liver Cancer; CA19-9, carbohydrate antigen199; CNLC, China liver cancer staging; Cr, creatinine; GGT, γ-glutamyl transpeptidase; HBV, hepatitis B virus; HCV, hepatitis C virus; INR, international normalized ratio; PLT, platelet count; PT, prothrombin time; PTA, prothrombin time activity; TB, total bilirubin.

**Table 2.** Tumor Response in the Total Cohort and Subgroups

	Total		*P*	BCLC B		*P*	BCLC C		*P*
	TACE-L (n = 51)	TACE (n = 51)		TACE-L (n = 15)	TACE (n = 16)		TACE-L (n = 36)	TACE (n = 35)	
CR	2 (3.9%)	3 (5.9%)	3	1 (6.7%)	3 (18.75%)	3	1 (2.8%)	0 (0%)	3
PR	15 (29.4%)	10 (19.6%)	3	6 (40%)	0 (0%)	3	9 (25%)	10 (28.6%)	3
SD	18 (35.3%)	12 (23.5%)	3	6 (40%)	7 (43.75%)	3	12 (33.3%)	5 (14.3%)	3
PD	16 (31.4%)	26 (51%)	3	2 (13.3%)	6 (37.5%)	3	14 (38.9%)	20 (57.1%)	3
ORR	33.33%	25.50%	.128	47%	18.75%	.161	28%	28.57%	.941
DCR	68.63%	49.10%	.044	86.70%	62.50%	.124	61.1%	42.90%	.124

Data are presented as n (%).

CR, complete response; DCR, disease control rate; L, Lenvatinib; ORR, objective response rate; PD, progressive disease; PR partial response; SD, stable disease; TACE, transarterial chemoembolization.

**Table 3.** Median OS and PFS in the Total Cohort and Subgroups

	Total		*P*	BCLC C		*P*
	TACE-L (n = 51)	TACE (n = 51)		TACE-L (n = 36)	TACE (n = 35)	
Median OS (months)	12.17	10.17	.042	11.5	8.03	.009
Median PFS (months)	3.33	2.9	.007	3.1	2.9	.012

OS, overall survival; PFS, progression-free survival; TACE, transarterial chemoembolization.

**Table 4.** Prognostic Factors for Overall Survival (Cox Hazard Analysis)

Factor	Overall Survival			
	Univariate Analysis		Multivariate Analysis	
	HR (95% CI)	*P*	HR (95% CI)	*P*
Age	0.988 (0.967-1.001)	.252	3	3
TB	1.003 (0.996-1.009)	.439	3	3
ALT	1.003 (0.996-1.009)	.352	3	3
AST	1.001 (0.999-1.003)	.351	3	3
ALP	1.02 (1.0-1.04)	.014	1.002 (1.000-1.005)	.039
Ca 19-9	1.001 (1.000-1.002)	.165	3	3
INR	1.871 (0.631-5.544)	.258	3	3
AFP	1.003 (0.996-1.009)	.05	1.002 (0.998-1.002)	.059
PT	1.013 (0.996-1.114)	.785	3	3
Hepatic vein invasion	2.340 (1.465-3.737)	.001	1.562 (0.821-2.972)	.174
Extrahepatic metastasis	1.477 (0.909-2.302)	.119	3	3
MELD	1.045 (0.987-1.107)	.134	3	3
Child‒Pugh	1.5 (0.942-2.394)	.087	0.703 (0.405-1.220)	.21
ALBI	1.46 (0.948-2.249)	.086	1.403 (0.805-2.443)	.232
BCLC	2.385 (1.370-4.154)	.002	0.601 (0.280-1.289)	.191
CNLC	1.477 (1.124-1.940)	.005	3	3
Treatment option TACE-L	0.629 (0.398-0.992)	.046	2.203 (1.321-3.676)	.002

AFP, alpha-fetoprotein; ALB, albumin; ALBI, albumin–bilirubin grade; ALP, alkaline phosphatase; AST, aspartate transaminase; BCLC, Barcelona Clinic Liver Cancer staging; Ca 19-9, carbohydrate antigen 19-9; CNLC, China liver cancer staging; GGT, γ-glutamyl transpeptidase; INR, international normalized ratio; PT, prothrombin time; TB, total bilirubin.

**Table 5.** Prognostic Factors for Progression-Free Survival (Cox Hazard Analysis)

Factor	Progression-Free Survival			
	Univariate Analysis		Multivariate Analysis	
	HR (95% CI)	*P*	HR (95% CI)	*P*
Age	1.001 (0.981-1.022)	.924	3	3
TB	0.999 (0.994-1.005)	.868	3	3
ALT	0.998 (0.991-1.004)	.465	3	3
AST	1.003 (1.0-1.006)	.055	1.001 (0.997-1.005)	.585
ALP	1.0 (0.998-1.002)	.892	3	3
Ca 19-9	1.001 (0.999-1.002)	.416	3	3
INR	1.536 (0.582-4.056)	.386	3	3
AFP	1.0 (1.0-1.0)	.521	3	3
PT	1.025 (0.938-1.12)	.591	3	3
Hepatic vein invasion	2.252 (1.432-3.456)	0	1.529 (0.802-2.851)	.182
Extrahepatic metastasis	1.110 (0.709-1.736)	.649	3	3
MELD	1.005 (0.952-1.06)	.864	3	3
Child‒Pugh	1.408 (0.905-2.192)	.129	3	3
ALBI	1.026 (0.71-1.484)	.891	3	3
BCLC	2.083 (1.273-3.408)	.003	0.651 (0.329-1.287)	.217
CNLC	1.265 (0.990-1.617)	.06	3	3
Treatment option TACE-L	0.571 (0.373-0.872)	.01	1.694 (1.097-2.615)	.017

AFP, alpha-fetoprotein; ALB, albumin; ALBI, albumin–bilirubin grade; ALP, alkaline phosphatase; AST, aspartate transaminase; BCLC, Barcelona Clinic Liver Cancer staging; Ca 19-9, carbohydrate antigen 19-9; CNLC, China liver cancer staging; INR, international normalized ratio; PT, prothrombin time; TB, total bilirubin.

**Table 6.** Adverse Events of Therapies

Adverse Events	TACE-LEN (n = 51)		TACE (n = 51)	
	All Grades	Grade 3/4	All Grades	Grade 3/4
	n (%)	n (%)	n (%)	n (%)
Decreased albumin	28 (54.9%)	0 (0%)	1 (1.9%)	0 (0%)
Hypertension	23 (45.1%)	13 (25.5%)	6 (11.8%)	0 (0%)
Elevated AST	21 (41.2%)	2 (3.9%)	20 (39.2%)	1 (1.9%)
Elevated TB	18 (35.2%)	1 (1.9%)	11 (21.6%)	1 (1.9%)
Decreased PLT	17 (33.3%)	6 (11.7%)	10 (19.6%)	3 (5.9%)
Elevated ALT	17 (33.3%)	2 (3.9%)	18 (35.3%)	1 (1.9%)
Decreased WBC	14 (27.5%)	0 (0%)	5 (9.8%)	0 (0%)
Diarrhea	12 (23.5%)	0 (0%)	6 (11.8%)	0 (0%)
Elevated GGT	11 (21.6%)	0 (0%)	4 (7.8%)	0 (0%)
Proteinuria	11 (21.6%)	11 (21.6%)	0 (0%)	0 (0%)
Prolonged PT	9 (17.6%)	0 (0%)	6 (11.8%)	0 (0%)
Decreased appetite	9 (17.6%)	0 (0%)	5 (9.8%)	0 (0%)
Fatigue	8 (15.7%)	0 (0%)	7 (13.7%)	0 (0%)
Bleeding (gingiva)	7 (13.7%)	0 (0%)	3 (5.88%)	0 (0%)
Hand–foot skin reaction	7 (13.7%)	0 (0%)	0 (0%)	0 (0%)
Elevated creatinine	6 (11.8%)	0 (0%)	0 (0%)	0 (0%)
Joint pain	4 (7.8%)	0 (0%)	7 (13.7%)	0 (0%)
Dysphonia	1 (1.9%)	0 (0%)	0 (0%)	0 (0%)
Edema	1 (1.9%)	0 (0%)	1 (1.9%)	0 (0%)
Constipation	1 (1.9%)	0 (0%)	3 (5.88%)	0 (0%)

ALT, alanine aminotransferase; AST, aspartate transaminase; GGT, γ-glutamyl transpeptidase; PLT, platelet; PT, prothrombin time; TACE, transcatheter arterial chemoembolization; TB, total bilirubin; WBC, white blood cell.
